# Genetic structure of introduced populations: 120-year-old DNA footprint of historic introduction in an insular small mammal population

**DOI:** 10.1002/ece3.486

**Published:** 2013-02-04

**Authors:** Siobhan Simpson, Nick Blampied, Gabriela Peniche, Anne Dozières, Tiffany Blackett, Stephen Coleman, Nina Cornish, Jim J Groombridge

**Affiliations:** 1 Durrell Institute of Conservation and Ecology, School of Anthropology and Conservation, University of KentCanterbury, CT2 7NR, United Kingdom; 2 JSPCA Animals' Shelter89 St. Saviours Road, St. Helier, Jersey, JE2 4GJ, United Kingdom; 3 Institute of Zoology, Zoological Society of LondonRegent's Park, London, NW1 4RY, United Kingdom; 4 Muséum national d'Histoire naturelle, Département “Ecologie et Gestion de la Biodiversité”, UMR 7204 “Conservation des espèces, restauration et suivi des populations”Case postale 53, rue Buffon, 75005, Paris, France; 5 The Department of Environment, States of JerseyTrinity, Jersey, JE3 5JP, United Kingdom

**Keywords:** Conservation, genetic structure, islands, population genetics, red squirrels, reintroduction

## Abstract

Wildlife populations have been introduced to new areas by people for centuries, but this human-mediated movement can disrupt natural patterns of genetic structure by altering patterns of gene flow. Insular populations are particularly prone to these influences due to limited opportunities for natural dispersal onto islands. Consequently, understanding how genetic patterns develop in island populations is important, particularly given that islands are frequently havens for protected wildlife. We examined the evolutionary origins and extent of genetic structure within the introduced island population of red squirrels (*Sciurus vulgaris*) on the Channel Island of Jersey using mitochondrial DNA (mtDNA) control region sequence and nuclear microsatellite genotypes. Our findings reveal two different genetic origins and a genetic architecture reflective of the introductions 120 years ago. Genetic structure is marked within the maternally inherited mtDNA, indicating slow dispersal of female squirrels. However, nuclear markers detected only weak genetic structure, indicating substantially greater male dispersal. Data from both mitochondrial and nuclear markers support historic records that squirrels from England were introduced to the west of the island and those from mainland Europe to the east. Although some level of dispersal and introgression across the island between the two introductions is evident, there has not yet been sufficient gene flow to erase this historic genetic “footprint.” We also investigated if inbreeding has contributed to high observed levels of disease, but found no association. Genetic footprints of introductions can persist for considerable periods of time and beyond traditional timeframes of wildlife management.

## Introduction

Wildlife populations have been relocated and translocated by people for centuries, both accidentally and intentionally (Atkinson 1973; Huxley 2003; Kolbe et al. 2008). Accidental movement of animals is most frequently a consequence of stowaways or escapees (Atkinson 1973; Weir and Grant 2005), whereas intentional movements can be either as introductions of wildlife to new areas, reintroductions to areas where they once previously occurred, or as restocking activities – augmenting existing populations with additional individuals (Shorten 1954; Biebach and Keller 2009; Grandjean et al. 2009). This human-mediated movement of individuals can disrupt natural genetic structure by altering patterns of genetic differentiation or gene flow that may have previously occurred. Such an alteration of gene flow can have negative consequences including isolating sub-populations causing inbreeding, or joining separate locally adapted populations causing individuals to become less fit in the alternate environment. Against this backdrop, islands can provide valuable safe havens for wildlife species whose mainland populations are threatened with extinction (Cowie and Holland 2006). Indeed, conservation biologists have increasingly begun to use islands as refuges on which to “maroon” vulnerable wildlife populations (Cade and Temple 1995).

For recently introduced populations, their genetic make-up and spatial distribution of genetic diversity depends on a number of factors, including the number of founding individuals and their evolutionary origin, relative breeding success between them during the initial phases of establishment, rate of increase in population size and subsequent expansion, and the extent of gene flow within the introduced population (Freeland 2005). Understanding how these factors determine patterns of genetic structure in introduced populations is important in a conservation setting, where such genetic footprints might unknowingly be generated by conservation managers and could persist for considerable periods of time (Biebach and Keller 2009).

Genetic footprints of past introductions have been demonstrated for a large number of continental-sized populations (Vonholdt et al. 2010; Zalewski et al. 2010; Ransler et al. 2011), but few have looked at genetic footprints following introductions to small islands. Miller et al. (2011) examined genetic structure within an introduced island population of egg-laying skinks (*Oligosoma suteri*) in the 15 years immediately following their reintroduction to Korapuki Island, New Zealand. However, many human-mediated wildlife introductions have taken place across much longer timescales, with many founded well over a century ago. Whether genetic footprints of such events persist across such time periods could have far reaching implications for the long-term management of wildlife. Islands provide ideal settings in which to study how introduced populations are founded and become established and consequently how patterns of genetic structure develop. Their isolated nature restricts the extent of natural colonization (Rocha et al. 2010), while their limited geographic size can act to restrain population growth and expansion.

Here, we apply these ideas to a recently introduced population of red squirrels (*Sciurus vulgaris*) on Jersey, to look for a historic genetic footprint of the introduction. We use mitochondrial DNA (mtDNA) sequence data and nuclear microsatellite DNA markers to trace the evolutionary origins of the founding individuals and examine current genetic structure in light of the known ecological history of the population over the past few hundred years. This introduced red squirrel population is an ideal system to interpret patterns of genetic structure because the history of the introduction is well documented and the island's ecological and land-use history over the past 200 years has remained relatively unchanged. Furthermore, the island has remained free of invasive North American gray squirrels (*Sciurus carolensis*) and so the negative effects of competition and diseases that they can bring have not had an impact on this red squirrel population.

The red squirrel population on Jersey is an example of a “marooned” population. They are believed to have been introduced from the South of England – where red squirrels no longer occur – to three locations in the west of Jersey in 1894 (Le Sueur 1976). Although not native to the island, red squirrels naturally occur just 22 km away in France, and anecdotal evidence suggests that they were also introduced to the island from France on several occasions (Le Sueur 1976). Given this evidence of a multiple-source introduction, we extended our genetic survey of Jersey red squirrels to include the two putative source populations from France and the South of England. While we might expect to see a historic genetic footprint of this introduction on Jersey, there may have already been significant admixing. The population is currently estimated to be between 600 and 1000 individuals and covers the whole island where suitable habitat exists, indicating that population growth and admixing could have erased any genetic signature left by the founding populations (States of Jersey 2005).

The same physical properties of isolation and restricted geographic size of Jersey, which make this red squirrel population ideal for studying the development of insular genetic structure, may also have led to genetic problems within the population. In particular, restricted population size brings with it associated problems such as elevated extinction risk as a consequence of increased levels of inbreeding, accumulation of deleterious mutations, and accelerated rates of loss of genetic diversity (Lynch et al. 1995; Keller et al. 2012). In addition, recently founded island populations are unlikely to have been founded by large numbers of individuals, and therefore this genetic bottleneck can allow deleterious alleles to become established within a population. Alongside these problems, island populations are acutely exposed to impacts of disease, a threat which can be elevated in inbred populations (Spielman et al. 2004). Intriguingly, an unusual trait of the red squirrel population on Jersey is an unprecedentedly high incidence of amyloidosis, a disease that can have many underlying causes (Merlini and Bellotti 2003). Amyloidosis is the miss-folding and deposition of normally soluble proteins within body tissues (Merlini and Bellotti 2003). When the progressive deposition of amyloid proteins occurs in vital organs, it ultimately leads to organ failure and death. Amyloidosis can occur in response to chronic immunological stimulation and can also be caused by a wide range of both ecological and genetic factors, as evidenced by studies on humans, cheetahs, and birds (Landman et al. 1998; Merlini and Bellotti 2003; Zhang et al. 2008a). Amyloidosis has been found in a wide range of vertebrate taxa including birds, fish, and mammals (Mashima et al. 1997; Landman et al. 1998; Zhang et al. 2008a). During the initial study period on Jersey from June 2007 to October 2009, 220 squirrels were examined for the presence of disease as part of the on-going disease monitoring study of the Jersey red squirrel population and 37 (16.82%) individuals were found to have amyloid protein deposits within one or more of their sampled tissues. One explanation for the high incidence of amyloidosis on Jersey could be the expression of deleterious recessive alleles as a consequence of inbreeding and genetic impoverishment caused by founder effects from the introduction. Support for this hypothesis comes from the only other case of amyloidosis in red squirrels, a possible mild case found in an individual from another island population – the Isle of Wight (V. Simpson, pers. comm.). To the best of the authors' knowledge, there are no other published reports of amyloidosis in free-living red squirrels, despite extensive disease surveillance in this species.

Consequently, island populations, and in particular those known to have been exposed to a bottleneck event at introduction such as the red squirrel population on Jersey, are an important focus for conservation genetic management. Indeed, this “refuge” population comprises a valuable part of the fragmented population of red squirrels remaining on mainland United Kingdom. Although the red squirrel population on Jersey has grown since its introduction 120 years ago to more than 600 individuals (States of Jersey 2005), the alarmingly high prevalence of amyloidosis has the potential to threaten the persistence of this population. Therefore, along with determining the extent of genetic structure within the population, there is also a need to examine whether there is an association between inbreeding and disease, and thus identify any genetic management needs. For this study, we use microsatellite DNA markers to quantify levels of genetic diversity and to infer individual-level and population-level inbreeding for two island populations of red squirrel, on Jersey and the Isle of Wight, and to compare these measures to the neighboring mainland-sized population of red squirrels in France. We then use these data to determine levels of genetic structure on Jersey and to look for associations between genetic diversity and prevalence of amyloidosis, with the aim of providing guidance for conservation and disease management strategies for this valuable but insular small mammal population.

## Methods

### Study site and species

Jersey is 22 km from mainland France, 160 km from mainland Britain, and has an island size of 116 km^2^. The predominant land use is farming, although there are a number of steep-sided valleys which are uncultivated, which along with thick roadside verges provide suitable habitat for red squirrels (States of Jersey Statistics Unit 2012). Despite the fragmented nature of their habitat, red squirrels are found over most of the island. Red squirrels are a small arboreal mammal, able to survive in most types of woodland, provided there is not too much competition from other species (Shorten 1954). Females are able to have two litters of young each year under favorable conditions, although this has not been documented in the Jersey population (Magris and Gurnell 2002). The young tend to disperse in the autumn to areas of suitable habitat, and are capable of moving large distances, but only do so when necessary and generally only move to the next unoccupied area of suitable habitat (Shorten 1954). The furthest distance recorded for squirrels on Jersey is 3500 m for two juveniles and 3200 m for an adult (Magris and Gurnell 2002).

### Samples

As part of the on-going Jersey Society for the Prevention of Cruelty to Animals (JSPCA) red squirrel disease monitoring program, dead squirrels were collected on Jersey by members of the public and taken to the JSPCA. For this study, 169 Jersey squirrels that presented dead or had died as a result of illness or injury from July 2008 to January 2011 were examined. For all individuals, a full post-mortem examination was carried out and tissue samples were taken for DNA, along with location details. Cause of death was attributed to one of three groups: ill health, predation/trauma, or road traffic accident. Sick (classified as “diseased”) individuals were those with evidence of clinical illness and “amyloid individuals” were those that were found to have amyloid deposits detected at histopathology within examined sampled tissues. Thirty-four samples from the Isle of Wight were collected on an ad hoc basis by wildlife and forestry personnel between 1995 and 2007. One hundred and ten samples from France were collected from individuals that were victims of road traffic accidents throughout northern and western France. Specimens were stored frozen at −20°C and tissue samples for DNA analysis were collected and stored in ethanol at −20°C.

### DNA extraction

DNA was extracted as described in Nicholls et al. (2000) with the following modifications: Approximately, twice the amount of tissue (2 mm^3^) was used, 500 μL of digestion buffer was used with 30 μg of Proteinase *K*, the pellet was washed twice in 70% ethanol and air dried until all ethanol was removed, and the DNA pellet was dissolved in DNA grade H_2_O rather than EDTA (Ethylenediaminetetraacetic acid) TE (Tris ETDA) buffer.

### mtDNA amplification and sequencing

A 500-bp fragment of mtDNA control region was amplified from 72 Jersey individuals and 10 Isle of Wight individuals using the primers Sq070F (Ogden et al. 2005) and RScont6 (Hale et al. 2004). These primers were selected from published primers to maximize fragment length. In each individual sample, the fragment was amplified in 50 μL reactions containing 1× *Taq* reaction buffer (16 mmol/L (NH_4_)_2_SO_4_, 67 mmol/L Tris-HCl), 1.5 mmol/L MgCl_2_, 0.1 mmol/L each dNTP, 0.2 mmol/L each primer, 0.002 U *Taq* (Bioline, London, U.K.) and 2 μL template DNA under the following conditions 94°C for 4 min, then 30 cycles of 94°C for 30 sec, 49°C for 30 sec, 72°C for 1 min with a final extension of 72°C for 10 min. Polymerase chain reaction (PCR) products were purified and sequenced in both directions by Macrogen Genomics Europe in The Netherlands. mtDNA control region sequences for 110 individuals from France were provided for this study by A. Dozières, of which five were resequenced to corroborate results.

### Microsatellite DNA amplification

In all, 169 Jersey, 50 French, and 34 Isle of Wight individuals were genotyped at 15 microsatellite markers. This study used a number of microsatellite markers previously developed for red squirrels: Scv1, Scv3, Scv6, Scv8, Scv9, Scv12, Scv13, Scv14, Scv16, Scv23, Scv32 (Hale et al. 2001) and Rsu3, Rsu4, Rsu5, Rsu6 (Todd 2000). These markers were chosen based on their reliable amplification in the target species. Scv10 was used initially, but was excluded because it was found to be in linkage disequilibrium with Scv23. Each marker was amplified in 10 μL reactions containing 1× *Taq* reaction buffer (16 mmol/L (NH_4_)_2_SO_4_, 67 mmol/L Tris-HCl), 1.5 mmol/L MgCl_2_, 0.1 mmol/L each dNTP, 0.2 mmol/L each primer, 0.002 U *Taq* (Bioline), and 0.4 μL template DNA under the following conditions 94°C for 4 min, then 30 cycles of 94°C for 30 sec, 56°C or 58°C for 30 sec, 72°C for 1 min with a final extension of 72°C for 10 min. All markers were multiplexed prior to analysis (see Table 1 for details). Fluorescently labeled PCR fragments were detected using an Applied Biosystems 3730 DNA Analyzer with GeneScan ROX-500 size standard (DBS Genomics, Durham, U.K.).

**Table 1:** Details of multiplex combinations, whether the multiplexing was done pre- or post-PCR, allele size ranges, and null allele frequencies within the Jersey population, number of Jersey individuals successfully genotyped (*n*), Hardy–Weinberg equilibrium test *P-*value (*P*_HWE_) (those with a * are significantly out of HWE at 0.01 level, – indicates that the test was not performed) for each population and number of alleles per locus (A), mean observed (H_o_), and expected (H_e_) heterozygosity in both the Jersey population and the published data (Todd 2000; Hale et al. 2001)

				Jersey				Published				P_HWE_		
							
Locus	Multiplex	Pre- or post-PCR	Allele size range (bp)	*n*	A	H_o_	H_e_	A	H_o_	H_e_	Null allele frequency	Jersey	France	IOW
Scv1	2	Pre	175–185	184	4	0.522	0.574	8	0.407	0.737	0.09	0.2406	0.1474	0.0866
Scv3	7	Post	198–222	181	7	0.425	0.486	12	0.478	0.873	0.17	0.421	0.9166	0.0321
Scv6	5	Post	186–201	204	9	0.49	0.524	6	0.429	0.783	0.05	0.0731	0.1024	0.0193
Scv8	3	Pre	192–201	204	5	0.466	0.514	5	0.348	0.655	0.12	0.4328	1	0.2341
Scv9	4	Pre	192–199	194	8	0.536	0.683	5	0.25	0.431	0.13	0.0053*	0.1215	0.016
Scv12	1	Post	188–199	199	5	0.497	0.544	2	0.394	0.454	0.11	1	0.3695	0.0092*
Scv13	2	Pre	165–181	206	3	0.388	0.479	3	0.697	0.603	0.18	0.6737	0.0265	–
Scv14	7	Pre	194–196	210	4	0.333	0.396	3	0.027	0.103	0.14	0.0163	0.2252	0.0001*
Scv16	6	Post	182–193	193	5	0.513	0.621	5	0.357	0.696	0.14	0.1089	0.4463	1
Scv23	1	Post	157–164	201	6	0.463	0.56	8	0.5	0.657	0.13	0.2303	0.5088	0.1309
Scv32	4	Pre	230–141	187	5	0.582	0.653	6	0.394	0.614	0.21	0.0255	0*	0.0418
Rsu3	5	Post	162–165	204	4	0.387	0.476	7	0.574	0.522	0.16	0.2136	0.3231	0.0003*
Rsu4	7	Pre	253–283	183	7	0.574	0.698	8	0.723	0.781	0.15	0.2484	0.6567	0.0139
Rsu5	3	Pre	132–141	206	5	0.335	0.369	7	0.455	0.393	0.25	1	1	1
Rsu6	4	Pre	121–126	213	3	0.399	0.429	4	0.365	0.273	0.06	0.4336	0.2881	1

### Analysis

#### DNA sequence data

All sequences were examined by eye and edited using FinchTV v1.4.0 (Geospiza Inc., Seattle, WA). Consensus sequences were created in Bioedit v7.0.9.0 (Hall 1999). Additional red squirrel control region sequences were downloaded from Genbank (Benson et al. 2012). Initially, all red squirrel control region sequences were included, but duplicates, or similar sequences (those that were in the same clade in the phylogeny) from the same location, were subsequently excluded, primarily to allow for more straightforward visualization of the resulting phylogeny. Sequences were aligned using ClustalX v2.1 (Larkin et al. 2007). Neighbor-joining Jukes–Cantor phylogentic trees were constructed using the Geneious tree builder and bootstrapped for 1000 replicates as implemented within Geneious v5.4 (Drummond et al. 2011). DNAsp v5 (Librado and Rozas 2009) was used to identify which haplotype each individual has. The haplotypes were mapped using ArcGIS (ESRI, Redlands, CA) to allow visualization of patterns of spread since introduction.

#### Microsatellite analysis

Genotypes were scored using Genemapper software v3.7 (Applied Biosystems, Foster City, CA). Observed and expected heterozygosities were calculated per locus in GenAlEx6 (Peakall and Smouse 2006), null alleles were checked for using Cervus (Marshall et al. 1998), significant deviations from Hardy–Weinberg equilibrium (HWE) were tested for using GenePop v4.0.10 (Raymond and Rousset 1995; Rousset 2008), and sex linkage was checked for by eye.

#### Analysis of genetic structure

To assess the genetic structure within the Jersey population of red squirrels, we used two programs; STRUCTURE v2.3.3 (Pritchard et al. 2000) and GENELAND v3.3.0 (Guillot et al. 2005a,b). STRUCTURE was run for 200,000 iterations after a burnin of 100,000 iterations, under the admixture model with allele frequencies both correlated and uncorrelated, for *K* values between 1 and 10, with iterations for each *K* run 10 times. The assignment values, log likelihood scores and Δ*K* (Evanno et al. 2005), were examined using STRUCTURE HARVESTER (Earl and vonHoldt 2011) to infer the optimal number of clusters. GENELAND was run 10 times for population min = 1 to population max = 10, each for 100,000 iterations with thinning every 10 iterations, for all combinations of correlated and uncorrelated allele frequencies and with spatial information – where the individuals were sampled from – either included or excluded. These combinations allowed the performance of the various models to be compared and selection of the most appropriate one.

To assess the level of introgression between the two introductions, *K* was set to *K* = 2 and run under the same parameters as above in both STRUCTURE and GENELAND, so that for each individual, the proportion of their genome that originates from each introduction can be quantified. The rationale behind setting *K* as 2 is that there were two separate populations when they were introduced, as documented by the Société Jersiaise at the time (Le Sueur 1976) and corroborated by our mtDNA results, and we are now interested in assessing how they have spread and introgressed (similar to Arteaga et al. 2011). Both STRUCTURE and GENELAND calculate the proportion of each individual's genome that comes from each source population. CLUMPP (Jakobsson and Rosenberg 2007) was used to obtain proportion averages across the multiple runs. Those with high a proportion of one population only are not very admixed while those with proportions closer to 0.5 show a mixture of ancestry. To establish if there is a geographic pattern to individual genome proportions, and thus to what extent the introductions are introgressing, pie charts of individual genome proportions were mapped using ArcGIS (ESRI). This was done using genome proportions from STRUCTURE under the admixture model with allele frequencies uncorrelated and MIGRPRIOR set to 0.4 to allow for significant introgression. To determine whether individual genome proportions differed for amyloid individuals alongside any geographic pattern, we plotted west-to-east (the orientation chosen to bisect the observed cline) the logarithmic proportion of each individual's genome estimated by the microsatellite data to be of English origin against geographic longitude. Subsequently, to establish if there was a difference in the proportion of an individual's genome that is of English origin between the amyloid and nonamyloid individuals, we performed a regression analysis on these data for the amyloid and nonamyloid individuals separately and then conducted a *t*-test of both regression lines to determine whether the slope of the line for amyloid versus nonamyloid individuals was statistically different.

### Quantifying genetic diversity and levels of inbreeding

Although marker-based estimates of levels of inbreeding are known to vary in terms of the degree to which they correlate with inbreeding coefficients obtained from pedigrees, in many situations, including this study, it is not possible to obtain pedigree data. However, an assessment can be made of the degree to which a microsatellite marker set is suitable for estimating inbreeding by calculating heterozygosity–heterozygosity correlations (Balloux et al. 2004). We used Rhh (Alho et al. 2010) to carry out a heterozygosity–heterozygosity correlation to establish if the marker set used in this study could be used as a reasonable proxy for a measure of individual inbreeding.

To estimate inbreeding coefficients for each genotyped individual, we used the Triadic Maximum Likelihood (TrioML) (Wang 2007). This measure accounts for genotyping errors and inbreeding, has a reduced sampling variance, produces results that are biologically constrained, and unlike many other maximum likelihood methods, does not overestimate relatedness (Wang 2007). In addition, by calculating Pearson correlation coefficients, we confirmed that this measure of inbreeding correlated with other individual measures of inbreeding, including methods-of-moments estimators (*r* = 0.375), regression estimators (*r* = 0.843), internal relatedness (*r* = 0.898), standardized heterozygosity (*r* = −0.821), and heterozygosity–homozygosity by loci (*r* = 0.848) (Ritland 1996; Coltman et al. 1999; Lynch and Ritland 1999; Amos et al. 2001; Aparicio et al. 2006). To obtain individual TrioML values, we used the program COANCESTRY (Wang 2011). TrioML values were normalized using a square root transformation to allow parametric tests to be carried out. All transformations, graphs, and statistics were carried out within the R statistical computing software (R Development Core Team 2009). A one-way analysis of variance (ANOVA) was performed to test for a difference in TrioML values between the populations, followed by a Tukey's test to establish which populations were significantly different from each other. To assess whether levels of inbreeding are significantly associated with the cause of death, presence of disease or presence amyloidosis in the Jersey population, one-way ANOVAs were performed.

Alongside individual measures of inbreeding, we also calculated population measures of inbreeding. As a population measure of inbreeding, unbiased expected heterozygosity was calculated using Genalex (Peakall and Smouse 2006) for each of the three populations and for the Jersey population for sample cohorts split into diseased/not diseased and positive/negative for amyloidosis. A Kruskal–Wallis ( K–W) rank sum test was conducted to establish if there were differences between the populations and cause of death in the Jersey population and Mann–Whitney *U* (M-W-U) tests were used to compare between diseased and nondiseased groups of individuals on Jersey. Kruskal–Wallis rank sum tests and Mann–Whitney *U* tests were carried out within the R statistical computing software (R Development Core Team 2009).

To examine the possibility that admixture could be influencing the presence of amyloidosis, a generalized linear model (GLM) incorporating the logarithmic proportion of an individual's genome, its longitude and an interaction between the two factors were carried out. As an additional assessment of the data to test for an association between genotype and amyloidosis, locus-by-locus tests were carried out using a chi-squared test for each allele at each marker to establish if any of the markers genotyped are associated with amyloidosis. A Bonferroni correction was applied to account for multiple tests.

## Results

### DNA sequence data

**Figure 1:**
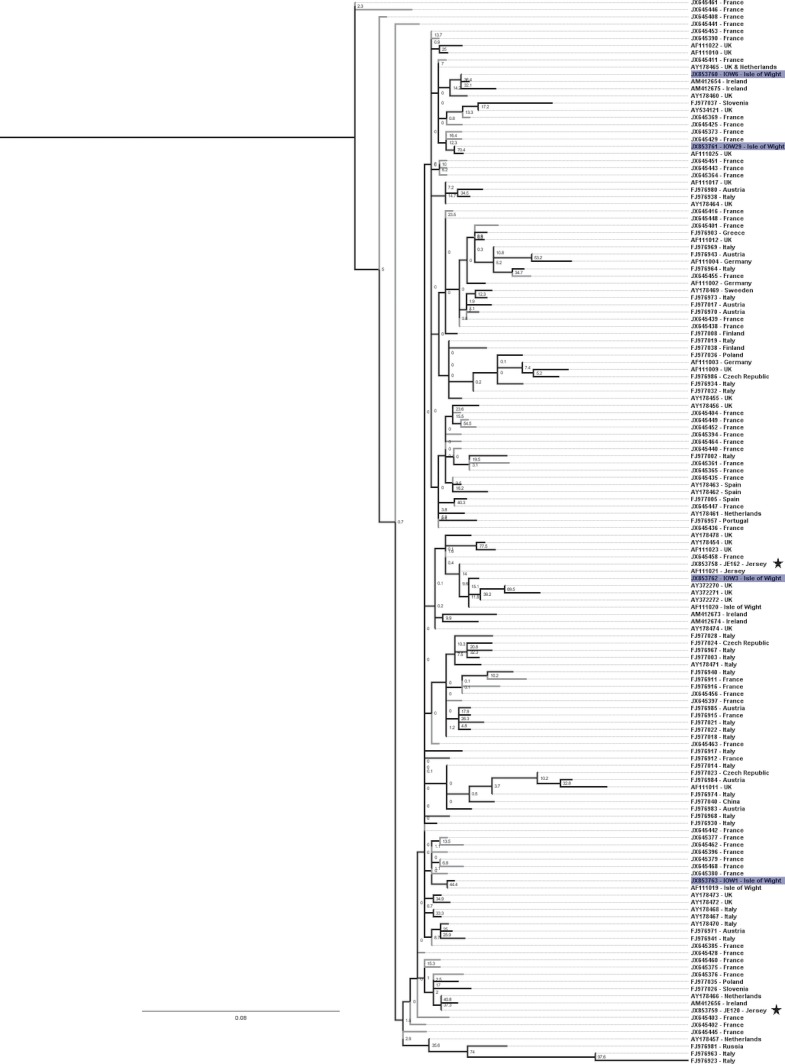
Neighbor-joining phylogenetic tree (Jukes–Cantor with 1000 bootstraps, rooted to *Sciurus vulgaris* (not shown). Nodes that include French haplotypes are gray, those that include haplotypes from Isle of Wight are shaded in gray and haplotypes from samples collected on Jersey are shown with a star. Sequences obtained from Genbank are annotated with their accession numbers, all have their country of origin in the name.

Only two mitochondrial control region haplotypes were detected on Jersey with eight variable sites between them (Genbank accession numbers JX853758–JX853759), indicating that they have separate evolutionary origins. The two Jersey haplotypes are located in two distinct clades, one is predominantly British (JE162), the other predominantly European (JE120) (Fig. 1). The phylogeny also illustrates that there are many haplotypes, which appear to be geographically out of place. Highlighted are the French haplotypes and the Isle of Wight haplotypes (IOW6, 29, 3, and 1) (Genbank accession numbers JX645360–JX645469 and JX853760–JX853763) to illustrate this point: both have their haplotypes located throughout the phylogeny, providing evidence of substantial movement of individuals by translocation and introduction throughout Europe.

**Figure 2:**
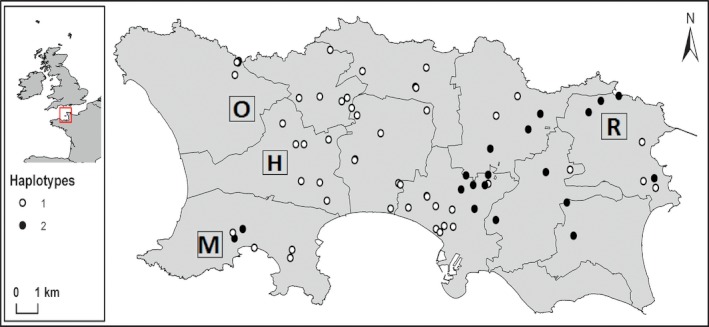
Map of location of the two mtDNA haplotypes found on Jersey. Haplotype 1 (white circles) is most similar to British haplotypes and haplotype 2 (black circles) is most similar to European haplotypes. The sites where red squirrels are documented to have been introduced are marked as follows: Rozel (R), La Hague Manor (H), La Moie House (M), and St Ouen's Manor (O).

The prevalence of the two haplotypes found on Jersey reveals that there is a geographic pattern to where the mitochondrial haplotypes occur on the island (Fig. 2). The British haplotype occurs mainly in the west of the island, while the European haplotype is predominantly found in the east.

### Microsatellite genotyping

A total of 169 individuals were successfully genotyped at most microsatellite loci. All microsatellite loci used were polymorphic and none had a null allele frequency greater than 0.25 (Table 1). There was some deviation from HWE, but it was not uniform across populations (Table 1) and so can thus be explained by population effects. In particular, Jersey is known to have had an unusual history of founder events and is a constrained island population, the Isle of Wight is also a constrained island population and there is limited information about the samples origin within the island – there may be substructure that has not been accounted for. Although France is not an island population, there may be substructure as the samples come from a wide range. Such founder events and genetic structure can cause deviations from HWE, so we chose to include markers that deviated from HWE provided that they did not consistently deviate across all populations.

### Analysis of genetic structure

The number of clusters that STRUCTURE and GENELAND infer varies from 1 to 9 depending on the input parameters. Applying an Evanno et al.'s (2005) correction to STRUCTURE output indicates that there are likely to be two or three fewer groups than the raw STRUCTURE output implies. However, this result varies according to the allele frequency model used. GENELAND also gives different results depending on the allele frequency model used and whether spatial data are included. Under the correlated allele frequency model, STRUCTURE gives nine clusters prior to correction and six afterward, GENELAND gives eight clusters when spatial data are excluded and four when spatial data are included. The uncorrelated allele frequency model gives fewer clusters, with STRUCTURE giving four prior to correction and two or four afterward, and GENELAND giving three clusters without spatial data included in the model and a single cluster when spatial data are included.

**Figure 3:**
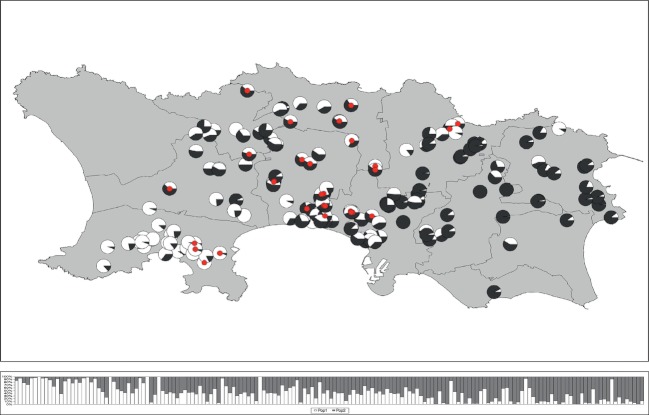
Upper panel – map of genome proportions from structure output when *K* = 2. Individuals with more white are those with more of their genome from group 1 and are found mainly in the southwest, while individuals with more black are from group 2 and are mainly found in the northeast. Those individuals with red dots were identified as having amyloidosis. Lower panel – the proportion of each individuals genome that is group 1 (white) and group 2 (dark gray) ordered by location from east to west.

**Figure 4:**
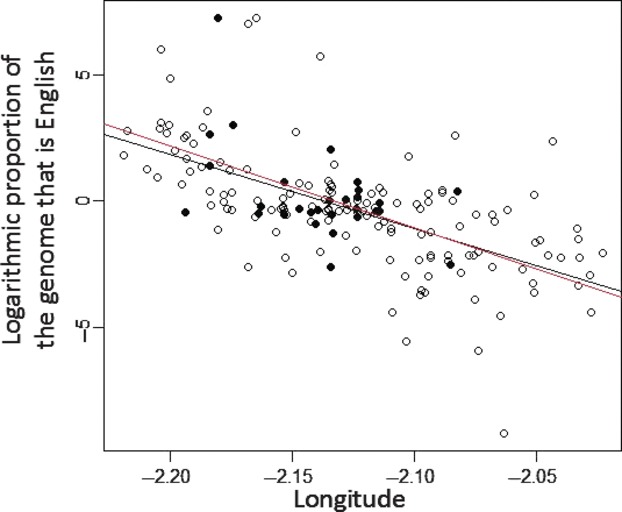
Plot of logarithmic proportion of an individual's genome that is estimated to be of English origin against geographic longitude of the sample location. Amyloid individuals are shown as black-filled circles; nonamyloid individuals are shown as unfilled circles. Lines are fitted for each group (red line, amyloid; black line, nonamyloid).

The uncorrelated allele frequency model and incorporation of spatial data appear to be the most realistic model for our data, based on our assumption that allele frequencies are unlikely to be correlated and the number and locality of clusters on the island. Therefore, to assess the introgression of the two introductions that we confirmed as having occurred using mtDNA haplotypes, the results from the uncorrelated allele frequency model are presented here. However, all of the model combinations under which both STRUCTURE and GENELAND were run gave broadly similar results. Figure 3 shows the proportion of each individual that is European and English in origin, individuals positive for amyloidosis are also indicated. There is an east–west pattern similar to that observed for the mtDNA haplotypes (Fig. 2). Figure 4 further demonstrates the east–west pattern, with the proportion of an individual that is English decreasing as they originate from further east.

### Quantifying genetic diversity and levels of inbreeding

When only 20 unrelated individuals were tested across all three populations, no single marker was consistently out of HWE, but when all individuals were included in an analysis, the Jersey and Isle of Wight populations were both significantly out of HWE (*P* = 0.001, with adjusted 5% level *P* = 0.00104), indicating that there is nonrandom mating occurring in these populations. The French population was within HWE, but only marginally so (*P* = 0.0021), suggesting some level of nonrandom mating.

We detected a significant heterozygosity–heterozygosity correlation (*r* = 0.1269; 95% confidence interval [CI] 0.03415, 0.22404), which indicates a consistency among the microsatellite loci for heterozygous individuals at one locus to be more likely to be heterozygous at other loci – a characteristic of the data set, which supports its use as a proxy for assessing levels of inbreeding among individuals.

We observed a significant difference in the levels of individual inbreeding coefficients between populations (ANOVA *F* = 5.39, *P* = 0.005), with both island populations having significantly higher mean levels of inbreeding than the mainland population in France (Tukey's Test Isle of Wight-France *P* = 0.015 and Jersey-France *P* = 0.008), but the level of inbreeding between the two island populations was not significantly different (*P* = 0.685). In contrast, levels of unbiased expected heterozygosity between the three populations were not significantly different (K–W = 3.9613, *P* = 0.138).

Tests among the individuals on Jersey for an association between inbreeding and cause of death, presence of disease, or amyloidosis revealed that there is no association between them when using either TrioML estimates (*F* = 1.1112, *P* = 0.3314; *F* = 1.1904, *P* = 0.2767; and *F* = 0.5574, *P* = 0.4563, respectively) or unbiased expected population heterozygosity (K–W = 1.0648, *P* = 0.5872; M-W-U = 111, *P* = 0.9674; M-W-U = 103.5, *P* = 0.7243, respectively).

A locus-by-locus approach to further examine the raw genotype data for an association between inbreeding and amyloidosis revealed that of a total of 122 alleles across the 15 different loci, three alleles (each at a different locus) showed a significant association with the prevalence of amyloidosis. However, after application of a Bonferroni correction, which decreased the significance level of the *P*-value from 0.05 to 0.000394, none remained significant.

The GLM indicates that the proportion of an individual's genome that is English is a significant factor (*P* = 0.0255) in whether an individual has amyloidosis, along with the interaction between the proportion of the genome that is English and longitude (*P* = 0.0260). However, comparison of regression lines between the two groups when the proportion of the genome that is English is plotted against longitude reveals that there is no difference between the groups (*P* = 0.7518).

## Discussion

Despite opportunities for admixture lasting well over a century, the population of red squirrels on Jersey has retained a phylogeographic genetic footprint of the introduction event that took place around 1894. This footprint is evident as genetic structure both in mitochondrial haplotypes and nuclear genotypes. The phylogenetic origins of the population concur with the historic records, which document that red squirrels were introduced to Jersey from both England and France over 100 years ago. Since then, levels of gene flow have not yet been sufficient to erase genetic structure completely. Our finding that a footprint of genetic structure can persist for so long has important implications for long-term management of small island populations. Although gene flow might be expected to occur – given enough suitably connected habitat – between individuals from different founder groups introduced to separate sites within a small island, the genetic footprint of an introduction can be surprisingly durable. Wildlife managers should not necessarily rely on the action of gene flow to disperse genetic diversity across populations on islands when planning management strategies for introduced or reintroduced populations.

### Phylogenetic origin

The two mitochondrial control region haplotypes occurring on Jersey are differentiated by eight nucleotide differences, suggesting that they came to be on the island as a result of two separate introductions, rather than as a consequence of mutation since the founding event. Introductions from multiple sources are not unusual for red squirrel populations. Previous genetic work has shown human-mediated movement of red squirrels to be extensive, particularly within the U.K. populations (Barratt et al. 1999; Hale et al. 2004; Ogden et al. 2005). This consistent human-mediated movement of red squirrels throughout their range can confuse signatures of introduction. However, our phylogenetic analysis (Fig. 1) does not refute information from historic records; red squirrels indeed appear to have been introduced to Jersey from both the United Kingdom and mainland Europe. One haplotype lies within a predominantly British clade – confirming the English introduction – while the other haplotype lies within a predominantly European clade that includes several French haplotypes, supporting anecdotal evidence of an additional introduction from France (Le Sueur 1976). Our phylogeny also illustrates the widespread human-mediated movement of red squirrels throughout Europe: the pattern of clustering and position of mtDNA haplotypes in Figure 1 does not fully correspond with location; for example, haplotypes from France and the four haplotypes from the Isle of Wight are distributed throughout the phylogeny.

### Extent of genetic structure

As the mtDNA data indicate that the Jersey population of red squirrels appears to have been founded from two evolutionarily distinct source populations (England and mainland Europe), it seems likely that strong genetic structure existed within the newly established population, a situation enhanced by the two founding events occurring at geographically separate introduction sites. However, how has this genetic structure changed over the last 120 years? Following establishment and a period of population growth, the extent of structure could have either remained the same (or increased) if no interbreeding occurred between the two differently sourced introductions, or diminished with interbreeding, potentially becoming erased entirely.

Remarkably, despite the length of time since the initial introductions for movement of individuals to have occurred, there remains a distinct east–west structure reflected in both mtDNA and nuclear markers that corresponds to historic accounts (Figs. 2 and 3). Historic documents suggest that English red squirrels were introduced to three locations in the west of the island (at La Hague Manor, La Moie House, and St Ouen's Manor; see Fig. 2) in 1894 (Le Sueur 1976), this is supported by our mtDNA results – the English haplotype is predominantly found on the west of the island. In contrast, the distribution of the mainland European haplotype is generally confined to the eastern half of the island, suggesting that red squirrels from mainland Europe were introduced into this area. This interpretation is also supported by anecdotal evidence suggesting that French squirrels were introduced to the Rozel area in the northeast of the island (Fig. 2) (Le Sueur 1976). Given that the mtDNA data reflect only female ancestry and movement, it would seem that females are slow to disperse across the island. The few mis-matched mtDNA haplotypes between the east and west sides of the island could be natural migrants, but more likely reflect recent human-mediated movements of individuals across the island, by the reintroduction of rehabilitated casualty individuals.

While the mtDNA loci revealed substantial genetic structure, the extent of structure revealed by nuclear loci was low. Our analyses using both STRUCTURE and GENELAND included specifying allele frequencies between clusters as being either uncorrelated or correlated. We ruled out the latter, having little reason to suspect allele frequencies between the two different source populations to be correlated. Furthermore, correlated models have been shown to be unreliable at resolving the true number of clusters even when the data are simulated under that model (Guillot et al. 2005a). However, different models using uncorrelated allele frequencies still gave varying numbers of clusters of between six and one. Part of the reason for this variation is likely to be the inability of Evanno et al.'s (2005) correction of STRUCTURE output to assess if the true number of clusters is one. However, we can assume that if there is genetic structure within the Jersey population, it would most likely occur spatially. Consequently, an uncorrelated allele frequencies model in GENELAND that included spatial data as a prior seemed to be the most appropriate model, the analysis of which returned a single cluster inferring that the population is panmictic. To find such little genetic structure within the population at the nuclear level, there must have been high a level of gene flow. Despite some studies reporting no difference between the movements of male and female red squirrels (Wauters et al. 2010, 2011), some find that males move further and more frequently (Shorten 1954; Lurz et al. 1997). As we have already established that females have not dispersed far, this nuclear gene flow is most likely facilitated by the movement of males. Indeed, a previous study of red squirrels on Jersey established that immigrants to three separate areas were biased toward males (Magris and Gurnell 2002).

Alongside this result of panmixia, however, we were also interested in assessing to what extent the two introductions have introgressed. Consequently, we reran STRUCTURE under the admixture model, allele frequencies uncorrelated, with prior information about population for those individuals that we obtained mtDNA genotype data for included and allowed for a high level of admixture by setting MIGRPRIOR to 0.4. Figure 3 illustrates a clear east–west split in the proportion of an individual that is English or mainland European in origin, a pattern which coincides with the east–west spatial pattern of mtDNA haplotypes. Our interpretation of this finding is that there has not yet been enough movement of individuals and gene flow across the island to completely erase a genetic footprint of the introduction events that occurred 120 years ago.

That genetic structure has remained intact for so long is perhaps due to the availability and connectivity of suitable habitat on Jersey. Land use on Jersey has remained relatively stable since the mid 1800s, with the majority of land used for farming and the remainder for human habitation. Despite a lack of extensive woodland on the island, those wooded areas that do exist are of high habitat quality for red squirrels, with a mixture of seed trees providing a reliable year-round source of food. However, there was a dramatic reduction in tree cover during the Second World War, before trees were rapidly replanted and regenerated in the post-war years, so that today the island contains many mature trees (Le Sueur 1976; Magris et al. 1997). In addition to these older trees, in recent years, there has been planting of new trees, particularly in hedgerows (States of Jersey 2005). Consequently, although this habitat is of good quality, much of it is highly fragmented which can reduce gene flow, yet since the recovery of wooded habitats, the red squirrel population has expanded naturally in the absence of competition from gray squirrels. However, explanations for the lack of association between inbreeding and the presence of amyloidosis are much less clear.

### Inbreeding and disease

Island populations are predicted to be significantly more inbred than mainland populations (Frankham 1998), and inbred populations (and individuals within populations) are predicted to be more susceptible to infectious diseases than noninbred populations (and individuals) (Wayne et al. 1991; Coltman et al. 1999; Acevedo-Whitehouse et al. 2003; Spielman et al. 2004). The first of these predictions appears to hold true for the red squirrel populations on Jersey and the Isle of Wight. Both these island populations are significantly more inbred than the mainland population of France according to individual inbreeding measures. However, to obtain these measures, we pooled data from across each population, which may have created Wahlund effects, causing us to conclude that a population is more inbred than it actually is. However, there is unlikely to be any Wahlund effect within the Jersey population as we have shown that it is one panmictic population. However, there is likely to be at least some action of the Wahlund effect within the French population as individuals came from a wide geographic area and therefore probably several sub-populations. Despite this effect, the Jersey population is still significantly more inbred than the French population, so this conclusion is valid. Unfortunately, we can be less certain about any Wahlund effect within the Isle of Wight population as we do not know whereabouts on the island sampled individuals were from and this may have inflated our inbreeding estimates. However, the area of the Isle of Wight is more similar to Jersey than to the size of the area where the French samples came from and there are many small well-connected woodlands on the Isle of Wight, so it is plausible that the population there is not sufficiently sub-structured to bring about a Wahlund effect. While not statistically tested, and although only one possible case of mild amyloidosis has been identified in red squirrels on the Isle of Wight, it could be assumed that both these island populations harbor individuals with amyloid deposits. However, within the Jersey population, those individuals estimated to be the most inbred appear to be no more likely to develop amyloidosis than less inbred individuals.

Although the population and individual measures of inbreeding from this study do not align regarding the difference in the levels of inbreeding between the three populations, both measures consistently find no association between the level of inbreeding and prevalence of amyloidosis or other diseases, therefore we must conclude that there is no association between inbreeding and amyloidosis or disease. Within any population, even those that are not inbred, there will be individuals that are more inbred than others and it has been shown that it is often these inbred individuals that are more susceptible to disease (Wayne et al. 1991; Coltman et al. 1999; Acevedo-Whitehouse et al. 2003; Spielman et al. 2004). Although our study was only based on 16 microsatellite loci, it may be considered unusual that there is no association between the level of inbreeding and amyloidosis. However, an individual does not have to be inbred to be predisposed to developing a disease, for example, heart disease in humans (Yiannakouris et al. 2012). Due to stochastic fluctuations in allele frequencies, certain populations may be more prone to disease than others. In the case of amyloidosis in red squirrels on Jersey, it may be that one or several of the original individuals carried an allele that conveyed susceptibility and through stochastic processes this allele has spread through the population. As our GLM indicates that the proportion of the genome that is English is a significant factor in explaining prevalence of amyloidosis, it may be that it was an individual of English origin that carried this susceptibility. However, for there to be an association between inbreeding and amyloidosis, the susceptibility allele has to be recessive. Consequently, individuals carrying such an allele do not have to be inbred to develop the disease, and therefore there may still be a genetic component to susceptibility to the disease, but inbreeding may not specifically enhance the likelihood of occurrence. Our locus-by-locus tests for association could have revealed such an allele if any of the loci genotyped were physically linked to genes that are important in determining susceptibility to amyloidosis. However, none of the associations were significant following a Bonferroni correction. This finding is not surprising because the markers we used were selected for their neutral properties. Alternatively, there may be very little genetic component to the disease, in which case inbred individuals would not be expected to have a greater chance of developing the disease. However, this scenario seems unlikely because amyloidosis is known to have a genetic component in many species, whereas in others, it is related to chronic infection, which may also have a genetic component (Merlini and Bellotti 2003; Zhang et al. 2008a,b). Evidence supporting the idea that there may be a genetic bias is the single case of amyloidosis outside of Jersey that has occurred on the Isle of Wight; individuals from the Isle of Wight are evolutionarily closest to the English haplotype found on Jersey (Fig. 1).

### Spatial distribution of amyloidosis

Despite not finding an association between individual genotype and amyloidosis within Jersey's red squirrels, our genetic analyses reveal an intriguing spatial pattern to the prevalence on the island. Of the 34 individuals found to have amyloidosis, most were found longitudinally down the center of the island – a distribution which broadly coincides with the area at the center of the island where our results suggest the two differently sourced introduced populations meet – a putative hybrid zone (Fig. 3). Outbreeding depression describes the situation whereby interpopulation hybrids experience lower fitness than their parent populations (Edmands 2007), a scenario which appears to fit that observed on Jersey if lower fitness in this case manifests as heightened susceptibility to amyloidosis. However, outbreeding depression has not yet been reported in red squirrels even though many populations are likely to have hybrid origins (Hale et al. 2004). In addition, we could not detect support for this hypothesis within our analyses. The *t*-test comparing regression lines of the proportion of the genome that is English against longitude found no difference between those individuals with amyloidosis and those without. The GLM also found no significant association between amyloidosis and locality, although it was significantly associated with the proportion of an individual's genome that is English. The finding that more individuals with amyloidosis have a higher proportion of English genetic origin could further support the hypothesis that there is a susceptibility allele within the population of English origin. However, we also note our finding that amyloidosis is significantly associated with the interaction between an individual's geographic location and the proportion that an individual's genome is English.

## Conclusions

Two introductions of red squirrels occurred on the island of Jersey, one from England and one from mainland Europe, likely France, comprising two evolutionarily different source populations, which most likely contributed to high initial levels of genetic structure. Our study has shown that at nuclear loci, this structure has subsequently diminished over the course of the past 120 years, largely as a consequence of male-mediated gene flow, but that it still remains detectable and reflects a genetic footprint consistent with the historic introduction events. Our results do not provide evidence that inbreeding has contributed to the unusually high prevalence of amyloidosis on the island, although this island population is significantly more inbred than the population on mainland France. The spatial distribution of those Jersey individuals with amyloidosis appears to coincide with a putative zone of mixing between east and west introduced populations, although we find no statistical support for any genetic association. Nevertheless, our findings advocate an important message to wildlife managers planning to introduce red squirrels or other small mammal species to island refuges – patterns of genetic structure caused by multiple introduction events can persist for considerable periods of time, well beyond the traditional timeframe of most population management plans.

## Data Accessability

DNA sequences: Genbank accessions JX853758-JX853763, JX645360-JX645469.

Microsatellite genotypes, sample locations, and amyloidosis status of Jersey individuals: Online supporting information.

## Supporting Information

**Table S1.** Microsatellite genotypes table for Jersey, French and Isle of Wight genotyped individuals..
